# MHC-I pathway disruption by viruses: insights into immune evasion and vaccine design for animals

**DOI:** 10.3389/fimmu.2025.1540159

**Published:** 2025-05-08

**Authors:** Yanan Wu, Zhuoya Sun, Lu Xia, Panpan Tian, Liuyang Jiao, Yanze Li, Zhanyong Wei, Xuannian Wang, Xiaoying Li, Gaiping Zhang

**Affiliations:** ^1^ College of Veterinary Medicine, Henan Agricultural University, Zhengzhou, China; ^2^ International Joint Research Center of National Animal Immunology, College of Veterinary Medicine, Henan Agricultural University, Zhengzhou, China; ^3^ Ministry of Education Key Laboratory for Animal Pathogens and Biosafety, College of Veterinary Medicine, Henan Agricultural University, Zhengzhou, China; ^4^ Longhu Laboratory of Advanced Immunology, College of Veterinary Medicine, Henan Agricultural University, Zhengzhou, Henan, China; ^5^ School of Advanced Agricultural Sciences, Peking University, Beijing, China

**Keywords:** MHC-I, adaptive immune responses, immune evasion, MHC-I assembly, MHC-I transport, MHC-I expression

## Abstract

Among various pathogens, viruses pose significant threats to the livestock and poultry industry, resulting in substantial annual costs due to production losses and vaccination. The MHC-I presentation pathway is a crucial surveillance mechanism for preventing viral infections. Consequently, many viruses have evolved sophisticated strategies to inhibit the presentation of viral peptides by MHC-I to CD8^+^ T-cells, thereby evading the immune system. Understanding the mechanisms that suppress the MHC-I pathway and identifying specific binding peptides are essential for comprehending viral immune evasion and developing effective animal vaccines. This review summarizes the viral strategies for evading immune recognition, including the inhibition of MHC-I molecules synthesis, degradation, transport, and assembly, which affect MHC-I surface expression during viral infections. We also present evidence that MHC-I surface expression is frequently lost during numerous viral infections in livestock and poultry and offer new insights into the underlying mechanisms through which viruses inactivate the MHC-I antigen presentation pathway. Collectively, these advanced findings on viral evasion from the MHC-I pathway could inform the development of more effectives strategies to restore immunological control over viral infections and improve vaccines for the livestock and poultry industry.

## 1 MHC-I is the key to promoting cellular immunity during antiviral infections

The Major Histocompatibility Complex (MHC) is a set of genes extensively studied for its critical roles in adaptive immunity. MHC initiates the adaptive immune response by presenting peptides to T cells across various pathological conditions, including pathogen infections, cancers, and autoimmune disease. MHC class I (MHC-I) molecules specifically present peptides to the T cell receptor of CD8^+^ T-cells, whereas MHC class II (MHC-II) molecules present peptides to CD4^+^ T-cells. Cytotoxic CD8^+^ T-cells are crucial components of the cellular immune response, playing significant roles in controlling viral infections. During viral replication, viral proteins are processed into small peptides by the proteasome. These peptides are transported to the endoplasmic reticulum (ER) lumen by the transporter associated with antigen processing (TAP), a member of the ATP-binding cassette (ABC) transporter family. Within the ER, virus-derived peptides may undergo further trimming by endoplasmic reticulum aminopeptidase 1 (ERAP1) and ERAP2 (1–3). Subsequently, these peptides associate with MHC-I molecules via the peptide-loading complex (PLC), which includes tapasin, TAP, calreticulin, and ERP57 (4). Alternatively, TAP-binding protein related (TAPBPR), not incorporated into the PLC, can also assist in this process (5). After assembly in the ER, MHC-I-peptide complexes travel to the Golgi apparatus and are transported to the cell surface via the secretory pathway, where they can be recognized by receptors on CD8^+^ T-cells, leading to apoptosis of infected cells (6). Both human and animal MHC-I contain α-chain + β2 microglobulins that form an antigen-binding groove and present endogenous short peptides (8-11aa), which deliver antigens to CD8^+^ T cells, triggering an immune response. Differences include extremely high HLA-I polymorphism (>20,000 alleles) in humans and lower in animals (e.g., mouse H-2), as well as a preference for hydrophobic peptides in human TAPs and positively charged residues in mice (7). The MHC-I-mediated antigen presentation pathway is a major component of antiviral immunity, thus protecting organisms against viral infections. MHC-I is a “safety label” for NK cells, and its normal expression inhibits NK cell activation, while its absence or abnormality triggers killing. The dynamic balance between the two maintains immune homeostasis and provides a key target for anti-tumor and anti-infection therapy (8).

## 2 MHC-I is the target molecule for virus escape

Given the pivotal role of MHC-I in antiviral immunity, it is unsurprising that the MHC-I-restricted antigen presentation pathway is a frequent target for viral immune evasion. This is evident from the close association between MHC-I surface expression and the adaptive immune response to virus-infected cells. Previous studies have reported that many viruses reduce MHC-I surface expression to evade T cell recognition (9–11). Mechanisms such as inhibition of proteasome function, TAP-mediated transport, retention of MHC-I in the ER, and interference with MHC-I synthesis and degradation regulate MHC-I surface expression. For instance, herpesviruses express numerous proteins that degrade MHC-I and inhibition TAP, thereby reducing MHC-I surface expression (12, 13). Influenza A or B virus infection causes a pronounced reduction in surface MHC-I expression in the late stages of infection through regulating proteasomal degradation and endocytosis of surface MHC-I, respectively (14). Viruses commonly manipulate multiple processes of the MHC-I presentation pathway to limit surface expression and evade immune recognition. This may explain why some vaccination programs fail to provide satisfactory protection against viral infection and clinical diseases. Elucidating the precise mechanisms underlying their interference with MHC-I dependent antigen presentation could be crucial for identifying responsible genes and designing improved vaccines. Designing vaccines can incorporate NK cell activation because NK cells are activated when MHC-I is down-regulated, so vaccines can be designed to stimulate both T cell and NK cell responses, such as adjuvants containing NK cell-activating ligands (15).

## 3 Viruses modulate the MHC-I processing pathway to achieve immune evasion

**Figure 1:**
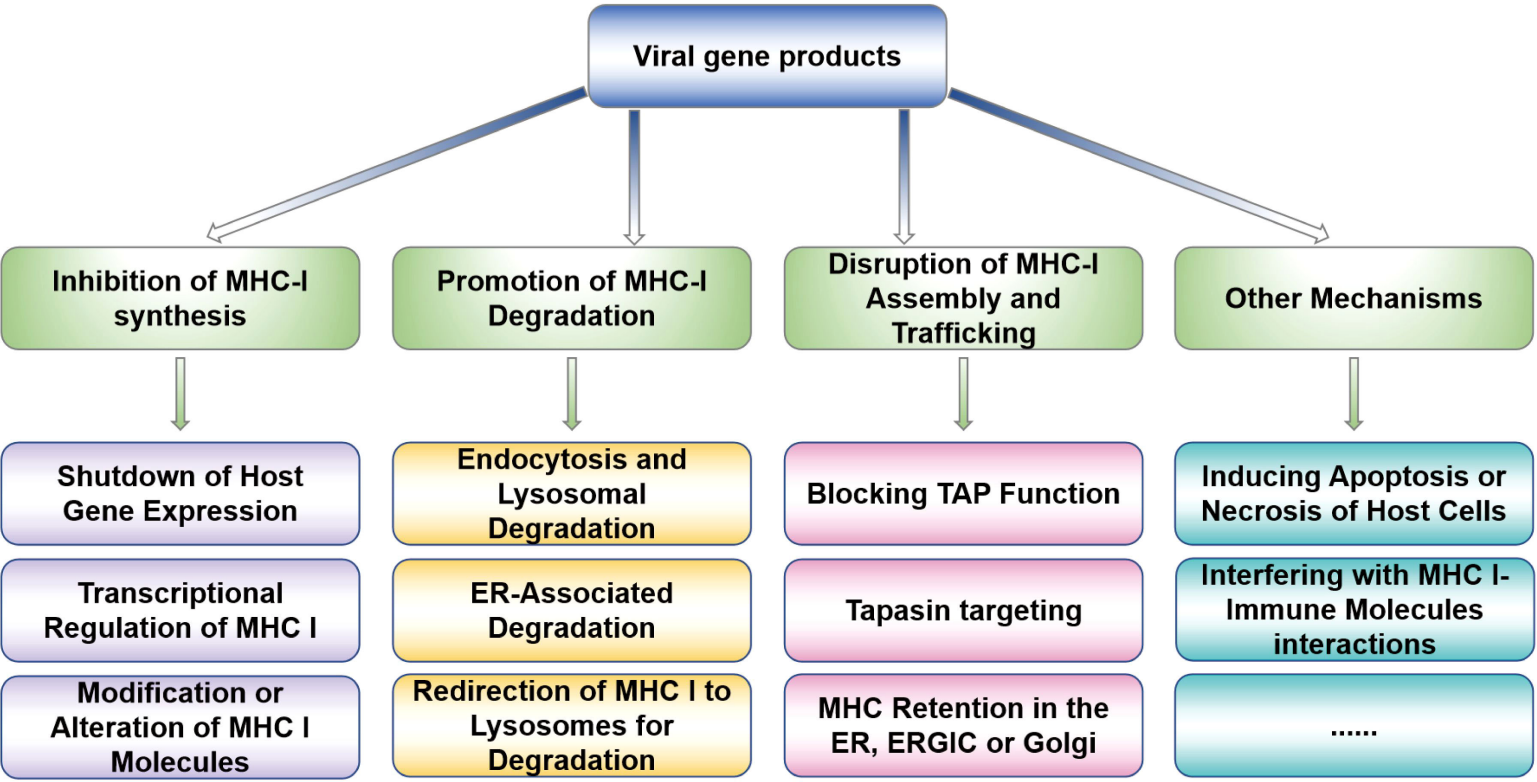
Viral strategies to prevent viral peptides presentation. The present diagram summarizes the viral strategies of evading MHC-I presentation, mainly including inhibiting MHC-I molecules synthesis and degradation, MHC-I transport, and MHC-I assembly to affect the MHC-I surface expression, which are frequent occurrence during viral immune evasion.

**Figure 2:**
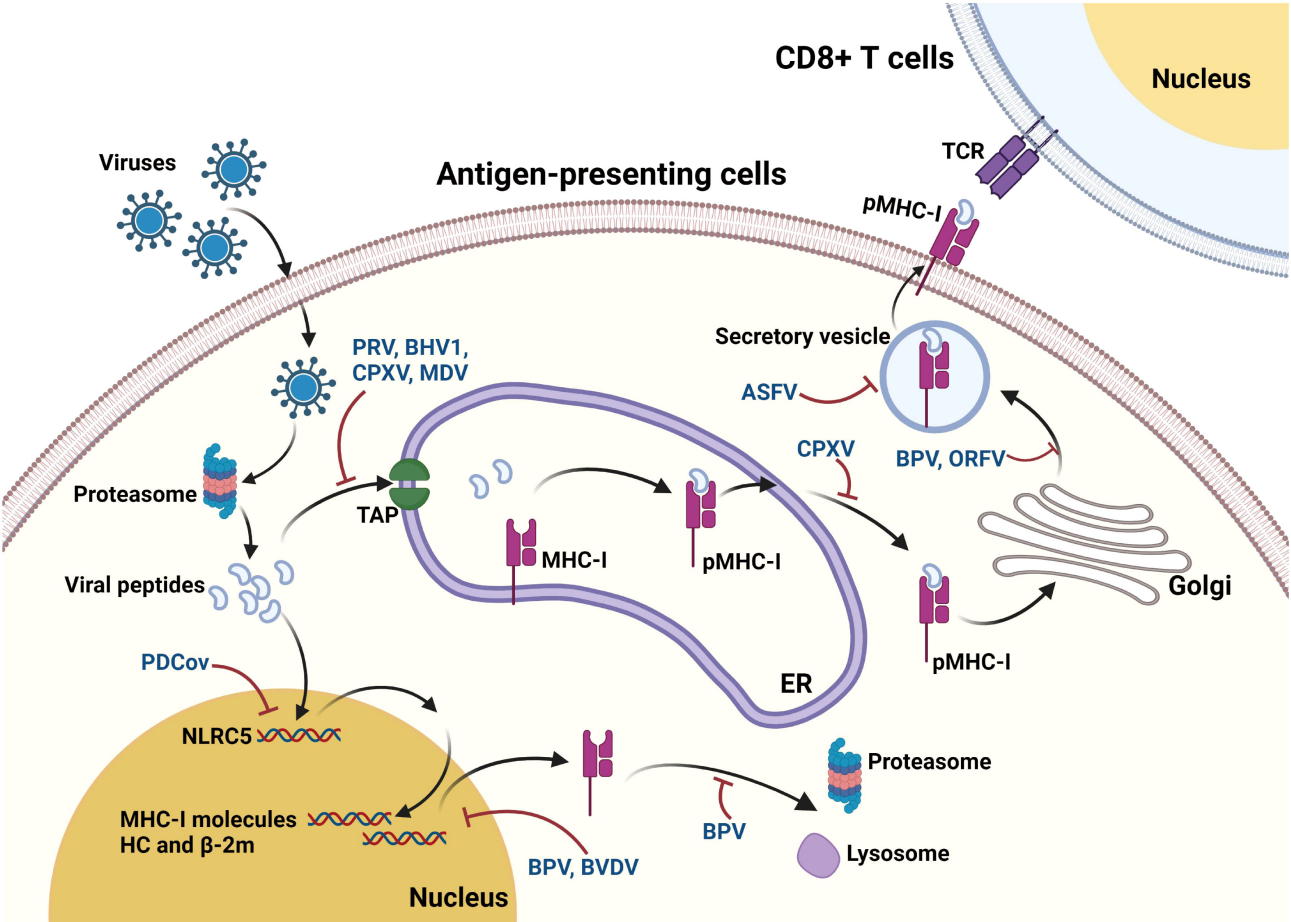
Mechanisms underlying the interaction between viruses and MHC-I pathway. After virus infection, viral proteins are processed into small peptides by the proteasome. The resulting viral peptides are transported to the lumen of ER through TAP. In the ER, the peptides are selected and then loaded on to the peptide binding groove of MHC-I complex to form the heterotrimeric MHC-I complex (pMHC-I). After assembly in ER, the pMHC-I then travels to Golgi apparatus and subsequently transport to the cell surface following secretory pathway, where they can be recognized by TCR on CD8^+^ T-cells. These viruses are commonly found to reduce the surface expression of MHC-I, while only a part of them are further identified to interfere with some events during the antigen processing. BPV suppresses the expression of MHC-I molecules, and also induces the proteasomal and lysosomal degradation of MHC-I molecules. Besides, BVDV causes reduction in expression of many proteins associated with MHC-I, endocytosis, and TAP. PRV, BHV1, CPXV, and MDV are demonstrated to inhibit the TAP functions to block peptides translocation into ER. CPXV causes retaining of MHC-I in ER, while BPV and ORFV causes MHC-I retaining in Golgi. ASFV impairs the exocytosis process of MHC-I to prevent the MHC-I membrane expression. Moreover, PDCoV upregulates the MHC-I surface expression via upregulating NLRC5 expression.

Viruses have evolved sophisticated strategies to prevent the presentation of viral peptides by MHC-I to CD8^+^ T-cells by co-evolving one or more gene products to interfere with MHC-I antigen processing. In the course of evolution, viruses have acquired abilities to regulate MHC-I degradation, inhibit MHC-I transcription, block TAP-mediated peptide transport, trap MHC-I molecules in intracellular compartments, interfere with chaperone-facilitated peptide loading, or rapidly reinternalize pMHC-I complexes, thereby evading the immune surveillance. In this part, we summarize the viral immune evasion mechanisms (**Figures 1**, **2**), including regulation of MHC-I synthesis and degradation, MHC-I transport, and MHC-I assembly. These insights contribute to the understanding how viruses modify MHC-I for immune evasion and aid in developing therapies and vaccines against viruses.

### 3.1 Regulation of MHC-I synthesis and degradation

#### 3.1.1 Inhibition of MHC-I synthesis by shutdown of host gene expression

Many viruses have been found to induce the shutdown of host gene expression, a strategy known as host shutoff, to modulate cellular machinery and evade host immunity (16). There is substantial evidence that some viruses can lead to dramatic downregulation of MHC-I expression at the cell surface through a general shutdown of host-cell protein synthesis (17). It has been reported that downregulation of MHC-I occurs as early as 3 h post bovine herpesvirus 1 (BHV1) infection and reaches a maximum level at 8 h post BHV1 infection, partly attributed to the virion host shut-off (vhs) protein (18).

#### 3.1.2 Transcriptional regulation of MHC-I genes

The tight regulation of genes encoding MHC-I is crucial for the adaptive immune response. NLRC5, a member of the NOD-like receptor (NLR) protein family, has been recently discovered to play a crucial role in the regulation of MHC-I transcription both *in vivo* and *in vitro (* 19–21). NLRC5 acts as a novel MHC-I transactivator (CITA) and forms an enhanceosome with the transcription factors at the promotor of MHC-I, such as the RFX complex, to induce MHC-I gene expression (22). These findings reveal that NLRC5-mediated expression of MHC-I molecules plays a vital role in modulating MHC-I antigen presentation. Recently, considerable evidence has shown that NLRC5/MHC-I transactivators constitute a target for immune evasion in cancer (23). However, little information is known about its association with viral immune evasion mechanisms, except for a study by Yoo et al, which finds that the SARS-CoV-2 ORF6 protein inhibits the induction of the MHC-I presentation pathway through direct suppression of the CITA function of NLRC5 via preventing NLRC5 nuclear importation (24).

#### 3.1.3 Viral modulation of MHC-I function through post-translational modifications

Viruses can alter the post-translational modifications (PTMs) of MHC-I molecules to disrupt their stability, trafficking, and antigen-presenting capacity. SARS-CoV-2 infection exemplifies this strategy by inducing allele-specific changes in the glycosylation patterns and abundance of human leukocyte antigen (HLA) class I molecules. Integrated immunopeptidomics and glycoproteomics analyses revealed that SARS-CoV-2 infection dynamically modifies the glycosylation o HLA proteins, particularly those associated with antigen-free intracellular pools. For instance: SARS-CoV-2 infection increases mono-glucosylated glycopeptides [e.g., Hex (10) HexNAc (2)] on HLA-C*15:02, indicative of improper folding and endoplasmic reticulum (ER) retention. These aberrant glycans are enriched in intracellular HLA pools, suggesting impaired trafficking to the cell surface (25).

#### 3.1.4 Regulating MHC-I endocytosis for internalization and lysosomal degradation

Endocytosis is a complex process, which is a process of transporting substances outside the cell into the cell through the deformation movement of the plasma membrane (26). It has been found that endocytosis plays a crucial role in the regulation of antigen presentation. Previous studies have shown that several members of alphaherpesvirus induce MHC-I downregulation through endocytosis. Human herpesvirus-8 (HHV8) has been shown to downregulate the cell surface display of MHC-I (27). To further identify the functional genes responsible for MHC-I downregulation, it was found that K3 and K5 enhance endocytosis and direct internalize of MHC-I molecules to endolysosomal vesicles for degradation (28). It has been found that infection with equine herpesvirus-1 (EHV1) results in enhanced endocytosis of MHC-I molecules from the cell surface of an equine skin fibroblast cell line, thereby leading to downregulation of MHC-I at the cell surface (29).

#### 3.1.5 Targeting MHC-I for endoplasmic reticulum-associated degradation

Endoplasmic reticulum-associated degradation (ERAD) is a conserved cellular quality control system that ensures the removal of misfolded or unassembled proteins from the ER. This process involves three key steps (1): recognition and tagging of aberrant proteins by chaperones and ubiquitin ligases, (2) retrotranslocation (dislocation) of the substrate from the ER lumen to the cytoplasm, and (3) proteasomal degradation following polyubiquitination. The ubiquitin-proteasome system plays a central role, as ERAD substrates are covalently modified with ubiquitin chains, which serve as signals for extraction and subsequent degradationER-associated degradation (ERAD) (30). Interestingly, some viruses hijack mammalian ERAD machinery to target MHC-I for proteasomal degradation in the cytoplasm (31). For example, human cytomegalovirus (HCMV) encodes glycoproteins US2 and US11, which redirect ERAD components to degrade major histocompatibility complex class I heavy chains (MHC-I HCs). These viral proteins bind newly synthesized MHC-I HCs in the ER shortly after infection, triggering their rapid ubiquitination. This step critically depends on ER-resident E3 ubiquitin ligases, such as TRC8 and TMEM129, which conjugate ubiquitin to MHC-I HCs (31). Following ubiquitination, US2/US11 facilitate the retrotranslocation of MHC-I HCs from the ER lumen to the cytoplasm—a process typically reserved for misfolded host proteins. Once in the cytoplasm, the MHC-I HCs undergo deglycosylation by N-glycanase (PNGase) and are rapidly degraded by the 26S proteasome. Remarkably, this viral strategy reduces the half-life of MHC-I HCs to 1–10 minutes, effectively preventing their cell surface expression and subsequent antigen presentation to cytotoxic T cells (32). These findings highlight the dual role of ERAD: while it primarily safeguards ER proteostasis, pathogens like HCMV co-opt this pathway to undermine adaptive immunity. Understanding how viral proteins interface with ERAD components provides insights into immune evasion mechanisms and potential therapeutic targets.

#### 3.1.6 Rerouting MHC-I to lysosomes for degradation

Human immunodeficiency virus (HIV)-1 Nef disrupts the transport of MHC-I by rerouting newly synthesized MHC-I from the trans-Golgi network (TGN) to lysosomal compartments for degradation (33). Adaptor protein (AP)-1 is a cellular protein complex that has been identified to be implicated in TGN to endolysosomal pathways. The ability of HIV-1 Nef to disrupt MHC-I trafficking is dependent on the expression of the mu1 subunit of AP-1 (34). It has been found that herpesviral protein pUL56 cooperates with pUL43 and participates in the downregulation of cell surface MHC-I to achieve immune evasion (35). The pUL43 is localized within Golgi vesicles, and lysosomes are responsible for degradation of pUL43. Cell surface expression of MHC-I is reduced in pUL43 and pUL56 co-expressing cells, and the rerouting of vesicles containing pUL43, pUL56, and MHC-I to the lysosomal compartment is observed (36). In addition, the transmembrane proteins pUL56 and pUL43 of Marek’s disease virus (MDV) interfere with the host’s MHC-I antigen presentation pathway through a synergistic effect, thereby suppressing the cellular immune response. Studies have shown that pUL56 promotes the degradation of host MHC-I molecules in lysosomes via a clathrin-dependent endocytosis pathway, while pUL43 may further inhibit the synthesis or surface expression of MHC-I (37). In summary, pUL43 and pUL56 drive the sorting of MHC-I for lysosomal degradation.

### 3.2 Viral disruption of MHC-I assembly and trafficking

#### 3.2.1 Retention of MHC-I in early secretory compartments

The ER-Golgi intermediate compartment (ERGIC) serves as a transient hub for cargo transport from the endoplasmic reticulum (ER) to the Golgi apparatus (38). Viruses exploit this pathway to block MHC-I surface expression. For instance, the m152-encoded gp40 protein from mouse cytomegalovirus (MCMV) binds MHC-I molecules, retaining them in the ER, ERGIC, or cis-Golgi via retrograde retrieval. Additionally, CPXV203 from cowpox virus sequesters MHC-I in the ER under Golgi-like acidic conditions by binding with pH-dependent affinity (39).

#### 3.2.2 Targeting the TAP complex for antigen processing disruption

The transporter associated with antigen processing (TAP), composed of TAP1 and TAP2, transports cytosolic peptides into the ER for MHC-I loading (40). Viruses disrupt TAP through diverse strategies. BoHV-1 UL49.5 triggers TAP degradation via the proteasome (41). Equine herpesvirus UL49.5 blocks ATP binding to TAP, halting peptide translocation (42, 43). Herpesviruses ICP47 binds TAP to inhibit peptide binding and ATP hydrolysis (44). Human cytomegalovirus (HCMV) US6 prevents ATP binding and conformational changes in TAP, blocking peptide transport.

#### 3.2.3 Inhibition of MHC-I assembly via tapasin subversion

MHC-I assembly in the ER requires tapasin, a chaperone within the peptide-loading complex (PLC) that stabilizes MHC-I and optimizes peptide binding. Viruses disrupt this process by different strategies. HCMV US3 binds tapasin to inhibit peptide loading, causing MHC-I retention in the ER. Molluscum contagiosum virus MC80 induces tapasin ubiquitination and degradation, leading to TAP loss and impaired peptide delivery. HCMV infection suppresses tapasin synthesis, destabilizing the PLC and MHC-I assembly.

### 3.3 Others

It appears that an ATP/ubiquitination/proteasome-dependent mechanism is responsible for the proteasome degradation of the majority of antigens presented by the MHC-I pathway. An increasing body of evidence indicates resistance of viral proteins to this proteasome degradation system. The Epstein–Barr virus (EBV)-encoded nuclear antigen (EBNA) 1 was shown to inhibit the presentation of MHC-I since it is resistant to ATP-dependent degradation (45). Thus, resisting proteasomal degradation may be another strategy for blocking the MHC-I pathway. Viral infections can induce host cell apoptosis or necrosis, which in turn affects the expression and function of MHC-I molecules. In a study by Wu et al (46), goose nephritic astrovirus (GNAstV) infection led to the degeneration and necrosis of splenic lymphocytes and renal epithelial cells. Conversely, during avian influenza virus (AIV) infections, the PB1-F2 protein of low pathogenicity H7N7 was found to restrict apoptosis in avian cells, thereby prolonging the survival of infected cells (47). Recent studies have also demonstrated that herpesviruses have evolved another immune evasion strategy: epitope evasion through the depletion of high-affinity peptides that fit into the MHC I binding cleft (48).

## 4 Interaction between MHC-I and viruses in livestock and poultry

Infectious diseases, caused by pathogenic microorganisms, are major factors limiting productivity and causing severe economic loss in the global livestock and poultry industry. Among these pathogens, viruses pose a significant threat to the livestock and poultry industry due to annual costs associated with production losses and vaccination. Viral infection triggers a series of innate and adaptive immune responses in the host. It is well established that MHC-I molecules play a crucial role in antiviral immunity by presenting intracellular antigens to CD8^+^ T-cells and enabling the elimination of virus-infected cells by natural killer cells. The CD8^+^ t cells are the core effector cells of adaptive immunity, functioning by specifically recognizing and killing abnormal cells, and the nk cells are part of the innate immune system, with a rapid, non-specific killing capacity, both of which have unique and complementary functions that are essential for protection against infection. Therefore, the identification of MHC-I binding peptides has been utilized in the design of peptide vaccines for therapeutic applications targeting pathogen-specific immunity in animals. MHC-I conjugate peptide vaccines have high safety and individualized therapeutic potential by targeting specific antigenic epitopes to activate CD8^+^ T cells, but their application is limited by insufficient MHC polymorphisms and immunogenicity, and they need to be combined with adjuvants, novel delivery technologies, or combination immunotherapies to enhance clinical efficacy. This chapter aims to give an overview of the mechanisms underlying the roles of MHC-I in antiviral immunity and viral immune evasion among viruses affecting livestock and poultry (summarized in **Table 1**). These data could advance our understanding of how these viruses manipulate MHC-I molecules and might help inform the development of therapies and vaccines against these viruses.

**Table 1:** Mechanisms underlying viruses interfering with MHC-I.

Virus	Functional proteins	Mechanisms of actions interfering with MHC-I	References
ASFV	EP153R	Impairing the exocytosis process of MHC-I	(58, 59)
PRRSV	unknown	Inhibiting the expression of MHC-I molecules	(51).
PDCoV	unknown	Inducing the expression of MHC-I molecules via NLRC5	(61)
FMDV	unknown	Regulation of MHC-I surface expression	(55)
PrV	one or more PrV early proteins	Interfering with the peptide transport activity of TAP	(56, 57)
BPV	E5	Regulating transcription of MHC-I heavy chain (HC); regulating degradation of MHC-I HC; causing retention of MHC-I in the Golgi apparatus	(63–65)
BHV1	UL49.5	Regulating transport of MHC-I molecules via inhibiting TAP functions	(43, 71)
BVDV	unknown	Inhibiting the expression of many proteins associated with MHC-I, endocytosis, and TAP	(74)
ORFV	unknown	Downregulation of MHC-I surface expression through disturbing carbohydrate trimming and maturation of MHC-I; causing structural disruption and fragmentation of the Golgi apparatus to impair the intracellular transport of MHC-I	(80)
CPXV	CPXV012	Inhibiting the TAP-mediated transport of antigenic peptides to interfere with the MHC-I assembly	(77)
	CPXV203	Interacting with MHC-I molecules and retaining them in the ER	(76)
MDV	UL49.5	Downregulation of MHC-I surface expression; interfering TAP degradation	(43)
CAV	VP2	Downregulation of MHC-I surface expression	(87)

### 4.1 Porcine reproductive and respiratory syndrome virus

PRRSV is one of the most relevant porcine pathogens worldwide, affecting the swine industry in most swine-producing countries (49). It has been documented that PRRSV can evade host defense mechanisms during the early stages of infection, as evidenced by a delay in the development of effective immunity in both infected and vaccinated pigs. To explore this mechanism, changes in phenotypic and functional properties of bone marrow-derived immature DCs (BM-imDCs) have been examined following PRRSV infection (50). The percentage of cells expressing MHC-I in BM-imDCs infected with PRRSV is significantly decreased compared to mock-infected controls at 48 h post-infection, along with an increase in the expression of CD80/CD86 on the cell surfaces. Thus, PRRSV infection results in the downregulation of MHC-I expression in BM-imDCs, which might help the virus evade detection and destruction by cytotoxic T-cells. Furthermore, PRRSV also causes a downregulation of MHC-I expression in lung dendritic cells (L-DCs) (51).

### 4.2 Foot-and-mouth disease virus

FMDV is a picornavirus that causes a highly contagious and rapidly spreading disease in cloven-hooved animals. Previously, researchers found that FMDV infection of epithelial cell lines, including porcine kidney cells (PK-15 and ESK-4), result in a rapid reduction of MHC-I surface expression to approximately 70% at 3 h post-infection, which renders the FMDV-infected cells unable to present viral peptides to T lymphocytes, aiding virus evasion (52, 53). FMDV has evolved to target epithelial cells as its main target for efficient replication and dissemination. FMDV-infected DCs have been demonstrated to induce the production of IFN-γ, which is an important regulator responsible for sustaining MHC-I presentation (54). Researchers found that the percentages of MHC-I increased in FMDV-infected DCs at 24 h post-infection (55). The ERK1/ERK2 pathway is involved in this modulation of MHC-I presentation.ERK1/2 regulates MHC-I antigen presentation at multiple levels, including gene transcription, antigen processing, interferon synergism, and endoplasmic reticulum assembly, and its activity directly affects the immune surveillance function of CD8^+^ T cells. Targeting ERK signaling may provide strategies to enhance anti-tumor immunity or control excessive immune responses. Besides, they discovered that inactivated FMDV (iFMDV) infection shows a relatively moderate upregulation of MHC-I molecules on DC membranes only after 2 h post-infection, which may explain the immune differences observed between infectious and inactivated particles.

### 4.3 Pseudorabies virus

Infection with PRV has previously been found to down-modulate the expression of MHC-I antigens in both murine and porcine cells (56). Further investigations have focused on the mechanism of MHC-I down-regulation. Researchers have confirmed previous findings, as evidenced by a progressive down-regulation of MHC-I expression in both PRV-infected PK-15 cells and PRV modified live virus (MLV) vaccine strains-infected PK-15 cells. Furthermore, inhibition of TAP activity in a dose-dependent manner is observed in PRV-infected PK-15 cells as early as 2 h post-infection, reaching a maximum level at 6 h post-infection. The results indicate that TAP inhibition may be one of the mechanisms underlying the down-regulation of porcine MHC-I molecules. They also found that one or more PRV early proteins, but not late proteins, are responsible for the down-regulation of MHC-I molecules (57).

### 4.4 African swine fever virus

ASFV is the sole member of the Asfarviridae family and belongs to the genus Asfivirus. It causes a highly lethal disease in domestic pigs, leading to substantial economic losses for the global pork industry. Several ASFV genes, including A238L, A179L, A224L, EP402R, and EP153R, have been identified as modulators of host defense mechanisms. Specifically, protein EP153R has been demonstrated to modulate the surface membrane expression of MHC-I antigens (58). Researchers have constructed a 3D model of the C-type lectin domain of EP153R and found that a dimer of this protein can asymmetrically interact with an MHC-I molecule to regulate its membrane expression. Further studies suggest that this modulation may occur through disruption of the exocytosis process (59).

### 4.5 Porcine deltacoronavirus

While MHC-I induction is typically associated with host antiviral responses triggered by cytokines such as interferons (60), its functional implications in PDCoV pathogenesis require careful interpretation. Mechanistically, PDCoV activates the RIG-I/IFN-βaxis, which subsequently elevates NLRC5 expression (61). As the major transactivator of MHC-I genes (CITA), NLRC5 forms an enhanceosome with RFX transcription factors to drive MHC-I promoter activity (60). Notably, PDCoV also enhances IRF1 expression, which directly stimulates NLRC5 promoter activity (61), creating a feedforward loop to amplify MHC-I synthesis. This pattern suggests two non-exclusive possibilities: As a component of host antiviral defense, elevated MHC-I may enhance viral antigen presentation to CD8+ T cells. Paradoxically, certain viruses exploit MHC-I upregulation to evade immune surveillance, such as through excessive self-antigen presentation that induces T cell exhaustion. While current evidence favors the first scenario due to the IFN-dependent pathway activation, PDCoV’s late-stage induction of MHC-I warrants further investigation into potential immune subversion strategies, particularly given that delayed MHC-I expression could allow viral immune evasion during early infection while promoting immunopathology at later stages.

### 4.6 Bovine papillomavirus

BPV is an oncogenic virus that induces papillomas in the cutaneous or mucosal epithelia of cattle. Over thirteen distinct BPV genotypes have been identified (62). BPV-4, one of the most extensively studies BPVs, has been linked to MHC-I modulation. The E5 oncoprotein, localized in the Golgi apparatus (GA) and ER during infection, plays a critical role in regulating MHC-I transport to the cell surface (63). Previous studies demonstrated that E5 physically interacts with the MHC-I heavy chain, reducing both total protein and mRNA levels in E5-transformed cells. The interaction between E5 and the MHC-I heavy chain can lead to reduced mRNA levels through several potential mechanisms. E5 may directly interfere with the transcription of the MHC-I gene by interacting with transcriptional regulators or modifying the chromatin structure, thereby decreasing mRNA synthesis (64). The physical interaction between E5 and the MHC-I heavy chain might disrupt the proper folding and processing of the MHC-I molecule, leading to its retention in the ER or GA (65). This retention could trigger cellular quality control mechanisms, such as the unfolded protein response, which may downregulate the expression of MHC-I at the mRNA level to prevent the accumulation of misfolded proteins. E5 might affect the stability of MHC-I mRNA by recruiting RNA-binding proteins or altering the cellular environment, leading to increased mRNA degradation. These mechanisms collectively contribute to the reduced mRNA levels of MHC-I in E5-transformed cells. However, treatment with IFN-β or IFN-γ can restore HC synthesis. Notably, MHC-I is retained intracellularly in E5-expressing cells, with its surface expression downregulated (63). Subsequent studies revealed that BPV E5 expression leads to MHC-I retention in the GA due to impaired acidification, preventing its transport to the cell surface (66). Moreover, E5-mediated inhibition of MHC-I transport is irreversible (67). In addition to inhibiting MHC-I HC transcription, E5 also promotes HC degradation, which can be reversed by treating with proteasome and lysosome inhibitors. Taken together, the BPV-4 E5 oncoprotein profoundly affects MHC-I expression, degradation, and transport.

### 4.7 Bovine herpesvirus 1

BHV1 establishes a lifelong infection in cattle, manifesting as infectious bovine rhinotracheitis and infectious pustular vulvovaginitis (68). BHV1 evades the host immune system by interfering with antigen processing and presentation. Following infection, MHC-I expression on the cell surface decreases as early as 8 h post-infection, reaching significant levels at 12 h. The immediate early and/or early proteins of BHV1 are implicated in mediating this effect (69). Besides, BHV1 impairs intracellular transport of MHC-I molecules and inhibits TAP in human melanoma cell lines (70). Comprehensive studies indicate multiple mechanisms contribute to BHV1-induced downregulation of MHC -I molecules (43). In particular, the BHV1-encoded UL49.5 protein, a homolog of the conserved glycoprotein N (gN), reduces MHC-I molecules on the cell surface and hinders their detection and lysis by cytotoxic T-cells. Several studies confirm that UL49.5 acts as a potent TAP inhibitor (43, 71). Based on findings from Wei et al. (72), specific residues in the UL49.5 luminal domain (residues 30-32) and CT region are crucial for promoting TAP inhibition and MHC-I downregulation.

### 4.8 Bovine viral diarrhea virus

BVDV is considered a group of multiple viruses affecting virtually all organs and systems in the body, including both innate and adaptive immune responses (73). Previous studies have reported that cytopathic (cp) BVDV infection alters professional antigen presentation in bovine monocytes (74). Notably, 9 MHC-I proteins are significantly downregulated in bovine monocytes after cp BVDV infection. Accession numbers AAZ73460 and ABA39524 have been identified only in cp BVDV-infected bovine monocytes. In addition, several proteins associated with endocytosis and TAP, such as small GTP-binding protein Rac1 and TAP-binding protein (tapasin), are abnormally regulated by cp BVDV, potentially contributing to the inhibition of their antigen presentation to immunocompetent T cells (74). However, the exact mechanism remains unclear.

### 4.9 Cowpox virus

CPXV is a member of the orthopoxvirus family that can infect a variety of mammalian species, with rodents serving as its dominant reservoir. Like other orthopoxviruses, CPXV encodes an array of immunomodulatory genes targeting antiviral responses. In numerous studies, CPXV has been established to disrupt MHC-I antigen presentation in both human and murine cells. Two unique CPXV-encoded proteins, CPXV012 and CPXV203, are responsible for blocking antigen presentation (75). The combined deletion of CPXV012 and CPXV203 restores the surface expression of MHC-I and stimulates CD8^+^ T-cells by CPXV-infected cells. CPXV203 is a soluble protein that binds MHC-I lumenal domains and exploits the ER retention pathway by a mechanism dependent on its C-terminal KTEL sequence, resembling the canonical ER retention motif KDEL recognized by the KDELR, thereby preventing MHC-I transport to the cell surface (76). CPXV012 is a short-lived type II transmembrane protein that co-immunoprecipitates with the PLC, inhibits TAP-mediated transport of antigenic peptides from the cytosol to the ER, thereby interfering with MHC-I assembly (77). Through these different mechanisms, CPXV adeptly evades immune responses.

### 4.10 Orf virus

ORFV is a zoonotic Parapoxvirus causing lesions in small ruminants, such as goat and sheep (78). It has been described that ORFV infection does not alter MHC-I transcription but disrupts carbohydrate maturation and maturation of the structural integrity of MHC-I molecules (79). ORFV also affects the Golgi apparatus’s fragmentation and structural disruption of the MHC-I molecule (80). Taken together, these findings impair the intracellular transport of MHC-I through these mechanisms to interfere with antigen presentation to immunocompetent T cells.

### 4.11 Marek’s disease virus

MDV is a herpesvirus that causes Marek’s disease, a severe neoplastic disease in chickens (81). Research has shown that MDV interferes with the expression of MHC-I molecules in chicken embryo fibroblasts (82, 83). This downregulation of MHC-I in MDV-infected cells can be counteracted by chicken interferon (IFN) produced by these fibroblasts (84). A recent study evaluated the role of MDV protein pUL49.5 in reducing MHC-I surface expression during MD pathogenesis. *In vitro* assays revealed that MDV pUL49.5 directly downregulates MHC-I in both transfected and infected cells (85). Previously, it was reported that the cytoplasmic tail of pUL49.5 plays an essential role in mediating this downregulation by targeting TAP degradation via the ubiquitin–proteosome pathway (43). Additionally, Jarosinski et al. (86) demonstrated that the cytoplasmic tail of MDV pUL49.5 significantly contributes to the downmodulation of MHC-I on the cell surface.

### 4.12 Chicken anemia virus

CAV is an immunosuppressive pathogen of chickens. Surface expression of MHC-I is markedly downregulated in cells infected with wild-type CAV. It is likely that viral protein 2 (VP2) is a multifunctional protein during CAV infection and replication. Site-directed mutagenesis of VP2 was designed to explore the role of VP2 in MHC-I regulation (87). The results show that mutations R101G and H103Y significantly abrogate the CAV-induced downregulation of MHC-I. Besides, mutation Q131P partially abrogate the effect of CAV on MHC-I downregulation. These findings confirm that MHC-I downregulation in CAV-infected cells is a virus-specific result and suggest that VP2 is an essential mediator of MHC-I downregulation. To date, the mechanistic events involved in MHC-I modulation remain unanswered.

### 4.13 Other viruses

CD8^+^ T-cells play a major role in the clearance of Respiratory Syncytial Virus (RSV) and mediate some of the damage to the lung epithelium following RSV infection. Interestingly, the expression of MHC-I is increased by human RSV (HRSV) infection in human respiratory epithelial cells and bronchial epithelial cells, which could be blocked by the addition of neutralizing Abs to IFN-β (in large part) and IL-1 α (a lesser extent) (88). Emerging evidence has shown that Bovine RSV (BRSV) shares many of the epidemiological and pathological features of HRSV in infants. Thus, some researchers speculate that MHC-I presentation may be altered by BRSV infection. Researchers have identified potential cytotoxic T-cells epitopes of BRSV, however, the regulation of MHC-I presentation by BRSV has not yet been elucidated (89). Besides, several other viruses such as Rous Sarcoma Virus (RSV) (90) and Duck Hepatitis A Virus type 1 (DHAV-1) (91) have been found to reduce MHC-I expression, although the mechanism remains limited.

## 5 Conclusions and perspectives

Due to the rising demand for animal-origin food, industrial livestock production has significantly intensified. This intensification has inadvertently facilitated the spread of various pathogens, predominantly viruses, leading to poor growth, mortality, and substantial economic losses, thereby posing major challenges. While vaccination remains fundamental to disease control, many existing vaccines are ineffective against certain viruses or emerging strains. Consequently, there is an urgent need to develop more efficacious vaccine products to better manage or prevent viral infections. Achieving this goal requires a deep understanding of the mechanisms by which viruses infect and evade immunity.

CTL immunity is crucial for host antiviral responses and plays a pivotal role in eliminating many viral infections. The antigen recognition and presentation via MHC-I molecules are integral to this response. MHC-I molecules select appropriate viral epitopes from infected target cells and present them to CD8^+^ T lymphocytes, prompting CTL cells to destroy virus-infected cells. However, viruses have evolved sophisticated strategies to evade immune surveillance, particularly by targeting MHC-I synthesis, degradation, transport, and assembly. These tactics effectively block the MHC-I antigen processing pathway, thus evading immune detection. Identifying viral epitope peptides and elucidating their regulation of MHC-I function are critical for understanding viral immune escape mechanisms and developing more effective vaccines. Several studies have shown that certain livestock and poultry viruses interfere with MHC-I expression, yet the molecular mechanisms by which these viruses down-regulate MHC-I remain largely unknown. Further research is needed to uncover how viruses regulate the surface expression and antigen presentation processes of MHC-I molecules. Unidentified functional proteins may also play roles in this process, and their discovery could significantly enhance our ability to develop vaccines that effectively prevent or control viral infections in livestock and poultry.

The potential for MHC-I to guide vaccine development is immense. By focusing on how viruses manipulate the MHC-I pathway, researchers can design vaccines that counteract these evasion strategies. For example, integrating peptides resistant to viral interference can enhance vaccine effectiveness. Additionally, understanding the precise points of viral intervention in the MHC-I pathway may aid in developing adjuvants that stabilize MHC-I expression, ensuring robust immune responses even during viral infections. Advances in bioinformatics and immunology have also paved the way for personalized vaccines. Tailoring vaccines to specific MHC-I haplotypes of individual animals can achieve higher levels of immunity, which is particularly beneficial for high-value breeding animals or endangered species.

To further enhance the effectiveness of MHC-I - based vaccines, one experimental approach could be to combine RIG - I agonists with the vaccine formulation. Since RIG - I plays a key role in the upregulation of MHC - I molecules as seen in the PDCoV example, activation of RIG - I by agonists may lead to increased MHC - I expression in vaccine - treated cells. This can potentially enhance antigen presentation and subsequent activation of CD8^+^ T lymphocytes, which are crucial for cell - mediated immunity against virus - infected cells. For example, small molecule RIG - I agonists could be co - formulated with the vaccine containing MHC - I epitopes (65). This could involve measuring the activation of CD8^+^ T cells, the production of cytokines related to cell - mediated immunity, and the overall protection of the animals against pathogen challenge.AI - driven epitope prediction offers a powerful tool to address this challenge in MHC - I - based vaccine design. Machine - learning algorithms can analyze large datasets of viral genomes and their corresponding MHC - I binding epitopes. By considering factors such as amino acid sequences, antigenicity, allergenicity, and toxicity, these algorithms can predict potential epitopes that are less likely to be affected by viral mutations. This has been widely used in the pre-preparation of vaccines for a variety of viruses (92, 93).

In conclusion, while viral infections pose significant challenges for industrial livestock production, the prospect of MHC-I-guided vaccine development offers a promising strategy to address these issues. Continued research into the molecular mechanisms of viral immune escape and the identification of novel functional proteins involved in MHC-I regulation will advance this field. As we gain a better understanding of these complex interactions, the path to developing more effective vaccines will become clearer, potentially enhancing disease control and increasing livestock productivity.
